# Protocol for evaluating microglial phagocytosis *in situ* in mouse hippocampal slices

**DOI:** 10.1016/j.xpro.2026.104696

**Published:** 2026-07-10

**Authors:** Summer G. Paulson, Fritz W. Lischka, Jeremy D. Rotty

**Affiliations:** 1 Uniformed Services University of the Health Sciences, Department of Biochemistry, Bethesda, MD 20814, USA; 2 Uniformed Services University of the Health Sciences, Department of Anatomy, Physiology, and Genetics, Bethesda, MD 20814, USA; 3 Uniformed Services University of the Health Sciences, Biomedical Instrumentation Center, Bethesda, MD 20814, USA; 4 The Henry M. Jackson Foundation for the Advancement of Military Medicine, Bethesda, MD 20817, USA

**Keywords:** Cell Biology, Microscopy, Neuroscience

## Abstract

Microglia are the primary phagocytes of the central nervous system (CNS). Here, we present a protocol for microinjecting opsonized particles into mouse hippocampal slices followed by two-photon time-lapse microscopy. We describe steps for preparing components, removing the brain, creating coronal slices, and incubating samples. We then detail procedures for analyzing microglial morphology and cell process dynamics with microinjected particles. We also provide several common potential problems encountered during the protocol alongside troubleshooting strategies to counter them.

For complete details on the use and execution of this protocol, please refer to Paulson et al.^1^

## Highlights

• Steps for visualizing microglia-bead interactions using time-lapse microscopy
• Instructions for quantifying microglial motility, morphology, and bead interactions
• Procedures for introducing small-molecule inhibitors into the slice workflow
• Guidance on navigating key potential problems and major troubleshooting strategies

## Abstract (editor highlights)

Publisher’s note: Undertaking any experimental protocol requires adherence to local institutional guidelines for laboratory safety and ethics.

## Graphical abstract

**Figure:**
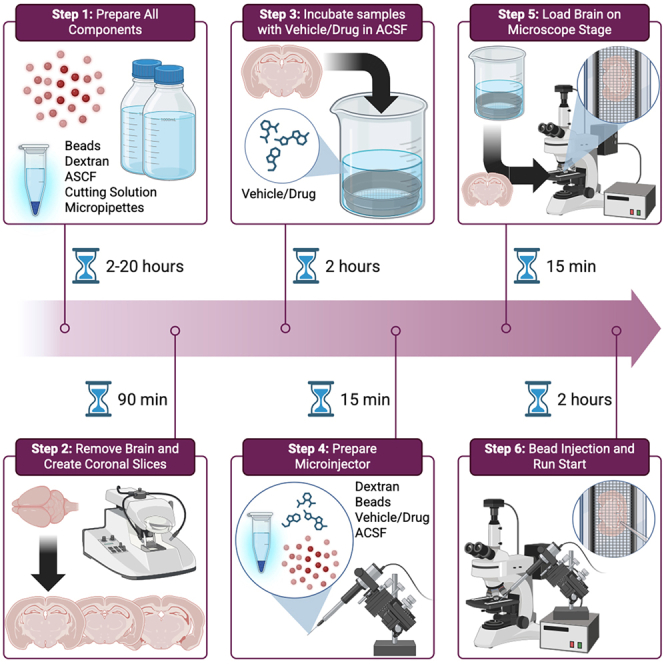

## Before you begin

The protocol below describes the injection of opsonized fluorescent beads into mouse hippocampal brain slices in which microglia are genetically labeled with GFP under control of the CX3CR1 promoter. The goal of this protocol is to examine microglial interaction with opsins and to interrogate phagocytic and haptotactic responses to opsins. This protocol can also be adapted to work with small-molecule inhibitors to target specific pathways or with chemotactic cues or other kinetic stimuli to quantify microglial response *in situ*.

### Innovation

While foundationally important, *in vitro* studies do not fully recapitulate the phagocytic processes occurring in complex *in vivo* microenvironments. For example, many studies are done with cells plated on surfaces far harder than physiological tissue,^2^ which lack the three-dimensional confinement found in tissues and do not contain other relevant microenvironmental cues that impact microglial function.^3^^,^^4^ These limitations could be circumvented by assaying microglia in their normal physiological niche, but it has traditionally been difficult to directly observe phagocytosis *in vivo* in the CNS.^5^ New technology such as Olympus’s scanR AI detection system allows scientists to bypass the need for exogenous dyes in live imaging, instead using this software to detect and track microglia in brightfield across live cell imaging videos and in fixed cell staining.^6^^,^^7^ However, this system is based on phase contrast images and most *in vivo* imaging requires fluorescent labeling to reach deep tissues, mark cells and provide enough contrast to detect cellular movement, which hinders the use of such approaches to investigate microglial function in the normal physiological niche. The use of imaging windows to assess microglial function in living, anaesthetized mice with GFP-expressing microglia has yielded important observations, especially when assaying microglial responses to laser ablation.^8^^,^^9^ However, it is difficult to introduce a defined cue (i.e., chemotactic or phagocytic ligands) in a controlled fashion using these *in vivo* strategies, so gleaning mechanistic insight into the pathways that regulate microglial sensing remains a challenge. Our approach overcomes these limitations by adapting previous workflows for *in situ* chemotactic and electrophysiological studies to observe microglia responding to phagocytic cues *in situ*.^10^^,^^11^ In the present protocol, we microinject opsonized targets (e.g., iC3b-labeled beads) directly into live mouse hippocampal slices, followed by timelapse two-photon microscopy, to quantify microglial interaction with targets. In addition to helping bridge the gap between *in vitro* and *in vivo* observations, a major strength of this protocol is its ability to quantify how microglia respond to phagocytic targets within their normal physiological microenvironment. We have also incorporated small-molecule inhibitors into our workflow, which enables mechanistic insights about microglial phagocytosis.

### Institutional permissions

An active mouse protocol was in place for the duration of these experiments. The mouse protocol was approved by the USUHS Institutional Animal Care and Use Committee (IACUC) prior to commencement of the studies related here, and in compliance with AVMA ethical standards.

### Solution preparation

**Timing: 1 h**

1. Prepare 1L of both Artificial Cerebral Spinal Fluid (ACSF) and Cutting Solution according to the recipes in the materials section.

**CRITICAL:** Take care not to mix measuring beakers, stir rods, or filter tops when making solutions, as these solutions should not be allowed to cross-contaminate each other.

2. Filter the solutions using a 0.22 μm pore bottle top filter, or similar, before storing them for consumption.

**CRITICAL:** Filtering the solution is required as even in well-dissolved solutions, small particulates may be present that will block the micropipette during injection.

***Note:*** Cutting solution requires 200 mL per mouse, so a 1 L solution will suffice for 5 mice. ACSF requires 750 mL-1 L per mouse, so a 1 L solution of ACSF should be made up for 1 mouse.

### Bead preparation

**Timing: 18 h**

3. Before opsonizing on beads, label 100 μg of iC3b with the pHrodo™ iFL Red Microscale Protein Labeling Kit according to kit instructions^12^ (https://documents.thermofisher.com/TFS-Assets/LSG/manuals/MAN0017102_pHrodo_iFL_Microscale_Protein_Labeling_Kits_UG.pdf).
  a. For iC3b, add 3 μL of the reactive dye to the protein. For other protein labeling, follow kit instructions to determine amount of reactive dye to add

***Note:*** This will yield approximately 100 μg of labeled iC3b, as each kit usage labels 100 μg of protein. This will last for roughly 15-20 injections. Typically, there are 3 injection conditions per mouse (non-treated, vehicle, and drug-treated), but additional beads may need to be made if there are issues with any of the original injections.

**CRITICAL:** The resulting solution should be a bright or dark pink. If it is nearly white, the dye has not labeled efficiently (**Troubleshooting Problem 1**). Repeat labeling protocol from the incubation step with an additional 3.5 μL of reactive dye, as recommended in the kit troubleshooting section. Column eluate should then be the correct color.

**CRITICAL:** Important steps to note when using the pHrodo iFL Red Microscale Protein Labeling Kit include the amount of pHrodo dye added to the protein for incubation, ensuring the eluate is the correct color to confirm labeling, and ensuring that the sodium bicarbonate does not pass a month of resuspension. The kit instructions state that sodium bicarbonate can be used up to 6 months when stored properly, but we found in our hands it performed less than ideally one month after reconstitution.

4. In a 1.5 mL Eppendorf tube, add the entirety of labelled iC3b, 30 μL of 2-micron Polybead Carboxylate Microspheres (hereafter referred to as beads), and 1 mL of 20–25°C PBS.
  a. Vortex Eppendorf tube.
  b. Wrap the Eppendorf tube in aluminum foil to protect the beads from light.
5. Place tube on rocker for 16–18 h at 4°C.
6. Centrifuge tube in a mini benchtop centrifuge at max speed (∼16,000 × *g*) for 30 seconds at 4°C.
  a. Aspirate old liquid out of Eppendorf tube.
  b. Resuspend iC3b bead mixture in 1 mL cold PBS.
  c. Centrifuge again at 16,000 × *g* for 30 seconds at 4°C.
  d. Repeat wash step twice more (3 total washes).
7. Resuspend beads in 30 μL of 20–25°C ACSF and store at 4°C away from light and/or wrapped in foil.

**Figure 1:**
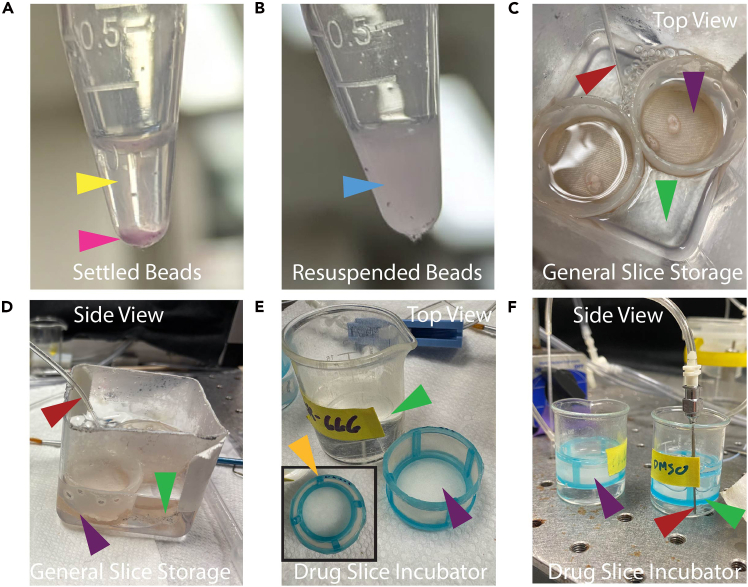
Before you begin preparation of materials (A) Image depicting pHrodo-labeled protein-opsonized beads (pink arrowhead) settled in the ACSF (yellow arrowhead) in a 1.5 mL microcentrifuge tube, either after the centrifuge step during washing or after long term storage at 4 °C. Note the pink coloration of the settled beads, marking the pHrodo-red label’s presence. (B) Image depicting the resuspended bead mixture after vortexing or rapid pipetting (blue arrowhead). Note the milky white coloring, as the pHrodo-red label is diluted in the ACSF. (C–F) Depictions of the tissue containers used for slice incubation and drug incubation of slices. These containers have two spaces - one part to hold the slices containing a mesh to allow ACSF exchange and one part that houses the source of the CO2 gas exchange in the ACSF. Purple arrowheads indicate where the mesh that the slices will sit on is present. This is either nylon stretched over the cups in the general slice storage or the bottom of the 40 μm filters used in the drug incubation storage. Green arrowheads indicate the space outside of the filtered area to accommodate the bubbler (as gas should not be bubbled directly onto the slices to preserve slice health). Red arrowheads indicate the gas line running the CO_2_ mix into the ASCF, to continue to preserve slice health. The orange arrowhead and inset in E demonstrates where the 40 μm filter’s edge has been cut so that it better fits into the 20 mL beaker. Seen in markings along the filter rim near the orange arrowhead is a label denoting which drug incubation this filter is associated with. As demonstrated in F, separate system set ups are needed per drug and vehicle incubation.

**CRITICAL:** When pelleted, the beads should appear pink (Figure 1A, pink arrow). When the beads are vortexed for resuspension in the ACSF, the solution will appear a diluted cloudy white (Figure 1B, blue arrow). If the resuspended beads are still bright pink after 3 washes, refer to troubleshooting problem 2.

**CRITICAL:** Beads will settle over time. Be sure to resuspend by vigorously vortexing before each use.

***Note:*** At the end of step 7, beads can be stored at 4°C for up to 12 months.

### Micropipette preparation

**Timing: 1 h**

8. To fabricate micropipettes for bead injections, pull borosilicate capillaries on a pipette puller.
  a. Pull pipettes for bead injection to a long narrow-tapered tip (about 1 cm long taper).
    **CRITICAL:** Choose parameters on your puller that create a long taper at the tip so that the pipette can fit beneath the microscope objective. Pulling the pipette to a desired shape depends on several factors. The freely downloadable Pipette cookbook From Sutter Instrument (Rev. E, 2025; https://www.sutter.com/hubfs/WEB%20-%20PDF%20Files/cookbook.pdf)^13^ can be used as a helpful tool to determine the most critical parameters. Individual settings will vary with heating filament shape and age, type of glass, tension of pulleys …). We pulled pipettes shaped like shown in chapter 8, using relatively high heat, high velocity and only 2 cycles.
  b. Bead injection tip openings should be about 5–10 μm. Break the newly pulled pipette back to about the desired tip diameter.
    ***Note:*** We found it most predictable to tap the tip against the heating coil of the fire-polishing instrument to break short sections off until the desired diameter is reached.
  c. Fire-polish tip to reach final opening size.
    ***Note:*** Micropipettes can also be pulled for other injection needs, such as ATP injections to measure chemotaxis. For this, a 1 μm tip diameter is needed instead.

### Tissue container preparation

**Timing: 45 min**

9. Make a mesh insert container to hold the brain tissue slices after sectioning (Figures 1C and 1D).
  a. Obtain a basin that fits roughly 100 mL of solution and fabricate two inserts that will hold the coronal brain sections.
    **CRITICAL:** The inserts should fit snugly inside the basin, so they stay suspended above the bottom of the container to allow the solution to circulate freely around the tissue.
  b. Glue pieces of nylon stockings over the bottom of the inserts to create a catch for the tissue.
    **CRITICAL:** Use glue that is insoluble in water.
    ***Note:*** Here it is suggested to use nylon stockings for the bottoms. Nylon mesh will also work.
    ***Note:*** Two inserts in the basin allow for sorting during slicing between good and bad slices, with space to separate tissue so slices do not overlap.
    ***Note:*** This piece can be made once and used repeatedly after rinsing with deionized water after use.
10. Make up drug and vehicle mesh containers to incubate slices in their respective drug cocktails (Figures 1E and 1F).
  a. Obtain 1 20 mL beaker per vehicle or drug component in the assay.
  b. Obtain a corresponding number of 40 μm pore size filters.
  c. Trim the lip off each filter with scissors until it fits into the beaker.
  d. Label each filter and beaker with their corresponding vehicle or drug.

***Note:*** These can be rinsed and reused if the corresponding filter stays with its beaker to avoid cross-contamination.

## Key resources table

**Table:** 

REAGENT or RESOURCE	SOURCE	IDENTIFIER
**Chemicals, peptides, and recombinant proteins**		

iC3b	CompTech	Cat#A115
CK-666	Abcam	Cat#Ab141231
Anhydrous DMSO	ThermoFisher	Cat#D12345
Cascade Blue labeled 3000 MW Dextran	ThermoFisher	Cat#D7132
Corning™ Cell Culture Phosphate Buffered Saline (1X)	Fisher Scientific	Cat#MT21040CV
NaCl	Sigma Aldrich	Cat#S9888
KCl	Sigma Aldrich	Cat#P3911
NaH_2_PO_4_	Sigma Aldrich	Cat#71496
MgSO_4_ · 7H_2_O	Millipore Sigma	Cat#230391
MgCl · 6H_2_O	Sigma Aldrich	Cat#M9272
NaHCO_3_	Sigma Aldrich	Cat#S6014
CaCl_2_ · 2H_2_O	Sigma Aldrich	Cat#223506
D-glucose	Sigma Aldrich	Cat#G7021
Sucrose	Sigma Aldrich	Cat#S0389

**Critical commercial assays**		

pHrodo™ iFL Red Microscale Protein Labeling Kit	ThermoFisher	Cat#P36014

**Experimental models: Organisms/strains**		

B6.129P2(Cg)-Cx3cr1^tm1Litt^/J Adult, Male (for breeding)	Jackson Laboratory	RRID:MGI:3580076
C57BL/6 Adult, Female (for breeding)	Jackson Laboratory	RRID:MGI:2159769
B6.129P2(Cg)-Cx3cr1^tm1Litt^/J x C57BL/6 Heterozygotes 35-55 day old, both male and female (for imaging)	N/A	N/A

**Software and algorithms**		

Zeiss Zen Black image acquisition software	https://www.micro-shop.zeiss.com/en/us/softwarefinder/software-categories/zen-black/	RRID:SCR_018163
Arivis	Zeiss	Vision 4D
Fiji ImageJ2	http://fiji.sc/	RRID:SCR_002285
ImageJ Manual Tracking plugin	https://imagej.net/ij/plugins/track/track.html	Included in Fiji ImageJ2
ImageJ Chemotaxis Tool plugin and stand-alone Ibidi Chemotaxis and Migration Tool	https://ibidi.com/chemotaxis-analysis/171-chemotaxis-and-migration-tool.html	Version 1.01 (ImageJ plugin) for quantification of manual tracking Version 2.0 (stand-alone) for histogram creation
MoltiQ	https://imagej.net/plugins/motiq	https://doi.org/10.1091/mbc.e21-11-0585
GraphPad Prism	https://www.graphpad.com	RRID:SCR_002798

**Other**		

2-micron Polybead Carboxylate Microspheres	Polysciences, Inc	Cat#18327-10
Borosilicate glass capillaries	Kimble	Cat#34500-99
Horizontal Puller	Sutter Instrument	P-80 or P-97
Zeiss 7MP Microscope with pulse infrared laser (Chameleon Vision2)	Microscope – Zeiss Laser – Coherent	
PLI-100 Pico-Injector (Pico Spritzer)	Medical Systems Corporation	
PatchStar Motorized Micromanipulator	Scientifica	
Leica VT1200S Vibratome	Leica	
40μm sterile cell strainer (blue)	Fisher	Cat#22-363-547
Millipore® Steritop® Vacuum Bottle Top Filter with 150 mL Funnel 45mm threading	Millipore Sigma	Cat#SC2GT01RE
Razor	Fisher	Cat#18-100-970
Spatula	VWR	Cat#82027-530
Dissection kit	Kent Scientific	Cat#INSMOUSEKIT
Sintered stainless steel aeration stone		

## Materials and equipment

### Equipment parameters

• We recommend that the vibratome being utilized has not been previously used with tissue fixing solution. PFA is difficult to remove from all surfaces and any remaining fixative will create a toxic environment for tissue slices, and they will not be as viable. If a dedicated vibratome for physiological slices is not possible, thorough cleaning the instrument with distilled H2O, 70% and 100% ethanol and an aldehyde scavenger (like N-acetylcysteine) could be attempted. In that case, microglia morphology and length of tissue storage without loss of mobility should be carefully evaluated.

### Recipes

**Table:** Artificial Cerebral Spinal Fluid (ACSF)

Reagent	Formula Weight	Final concentration (in mM)	Amount (in g/L)
NaCl	58.44	126	7.36
KCl	74.55	3	0.22
NaH_2_PO_4_	120	1.25	0.15
MgSO_4_ · 7H_2_O	246.5	2	0.49
MgCl · 6H_2_O	203.3	2	0.41
NaHCO_3_	84.01	26	2.18
CaCl_2_ · 2H_2_O	147	2	0.29
D-glucose	180.2	10	1.80
Sucrose	242.3	20	6.85
ddH_2_O	N/A	N/A	Up to 1L
**Total**	**N/A**	**N/A**	**1L**

Store at 4°C for up to 3 months.

**Table:** Cutting Solution

Reagent	Formula Weight	Final concentration (in mM)	Amount (in g/L)
KCl	74.55	2	0.15
CaCl_2_ · 2H_2_O	147	1	0.15
NaH_2_PO_4_	120	1.25	0.15
MgSO_4_ · 7H_2_O	246.5	2	0.49
MgCl · 6H_2_O	203.3	2	0.41
NaHCO_3_	84.01	26	2.18
D-glucose	180.2	10	1.80
Sucrose	242.3	206	70.51
ddH_2_O	N/A	N/A	Up to 1L
**Total**	**N/A**	**N/A**	1L

Store at 4°C for up to 3 months.

### Alternative reagents

• Drug of choice for treating tissue samples (e.g., CK-666 for disrupting Arp2/3 complex) and vehicle control (e.g., DMSO). Other drugs and their respective vehicle controls can also be substituted here, depending on the scientific questions asked.

***Note:*** Conduct pilot experiments to ensure that the drug dose is enough to elicit a response but not so much that it or the vehicle affects cellular health. This will be further referenced in the main steps below, as well as in troubleshooting problem 13.

• We also used this system to inject ATP (Sigma Aldrich Catalog # A2383-5G) to examine chemotaxis. Other cellular responses might also be assayed using this setup.

***Note:*** When dissolving a drug (i.e. the Cascade Blue Dextran, powered ATP, etc.), ensure that you spin down the solution and only draw out the supernatant. This will be further referenced in the main steps below, as well as in troubleshooting problem 4.

## Step-by-step method details

### Brain slice creation

**Timing: 90 min**

This process removes the brain from the mouse, places it on the chuck for vibratome sectioning, and facilitates transferring viable brain tissue slices from the vibratome to a recovery solution of warm, bubbled ACSF for incubation.

**Figure 2:**
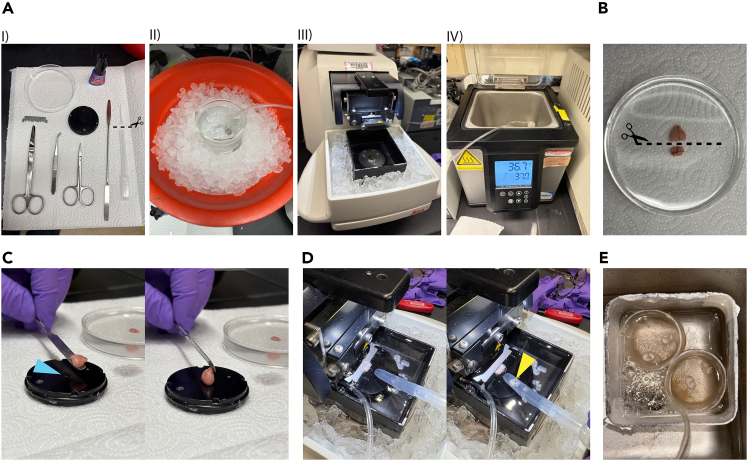
Workflow for hippocampal slice production (A) Preparation prior to removing the brain from the mouse. All tools should be laid out (i), cutting solution should be bubbled on ice (ii), the vibratome should be switched on, assembled, and cooling (iii), and the recovery ACSF should be bubbled at 37°C awaiting slices (iv). (B) The brain is placed in the Petri dish of pre-chilled cutting solution, and a razor blade is used to remove the cerebellum (dotted line). (C) Using a spatula, the brain is carefully flipped onto a dot of superglue on the vibratome chuck (marked in blue arrowhead). (D) Chuck is mounted onto a vibratome, and the brain undergoes coronal slicing. 400 μm thick slices are removed via transfer Pasteur pipette (yellow arrowhead) and moved to the container of 37°C ACSF in the water bath. (E) Slices incubate at 37°C in ACSF for 30 min.
2. Euthanize the mouse via CO_2_ exposure and remove the brain.
  **CRITICAL:** Brain removal needs to happen rapidly after the animal is euthanized to ensure maximum cell viability in tissue slices. Be sure to still perform secondary measures (i.e. toe pinch) to ensure lack of animal response.
  ***Note:*** Use care with brain removal. Nicks in the cortex during removal can lead to further separation and tearing of the tissue during subsequent handling and complicate secure mounting in the imaging chamber, causing tissue to drift during slice imaging.
  a. Place the brain in the prepared petri dish filled with cutting solution
  b. Place a dot of superglue onto the vibratome chuck
    ***Note:*** This is done at this time point to give the superglue time to become slightly tacky, but not so early that it is no longer adhesive when mounting the brain (troubleshooting 3).
  c. Take a razor blade and remove the cerebellum from the brain in a straight line (Figure 2B)
    ***Note:*** This is to set up for mounting the newly cut posterior end of the brain onto the vibratome chuck for coronal slicing.
3. Scoop brain tissue onto spatula end and mount the cut side of the brain onto the spot of superglue on the vibratome chuck, so that the olfactory bulb is facing up (Figure 2C).

***Note:*** Tap the brain on the spatula briefly to a paper towel before mounting to remove excess moisture from the brain, improving the bond between tissue and chuck.

1. Prepare workspace for brain slicing (Figure 2A).
  a. Set out each of your tools that you will need for this process.
    ***Note:*** These items will include scissors to remove the mouses head, scissors to open the head and expose the brain, a razor, superglue, a Pasteur pipette, a petri dish, and a spatula for removing the brain from the skull.
    ***Note:*** Tools used to remove the head and brain from the mouse may differ from experience preference. We suggest the tools that we found most useful (two different sized scissors and a spatula), but others may find other tools necessary in addition or in place of these, such as a forceps - which is shown in Figure 2A.
  b. Add 200 mL of cutting solution to a beaker placed in a bucket of ice.
  c. Add roughly 100 mL of ACSF to the mesh insert container placed in a 37°C water bath.
  d. Add an aeration stone attached to a 95% O_2_/5% CO_2_ gas mixture source to both the cutting solution containing beaker and the ACSF containing container to condition the solutions with the gas mixture.
  e. Cut off the tip of the Pasteur pipette close to the ridge on the barrel as indicated in Figure 2A.
    ***Note:*** Cutting off the tip of the Pasteur pipette is done to create a larger opening to accommodate the transfer of the coronal mouse brain section.
  f. Add a razor blade to the vibratome.
  g. Immediately preceding mouse dissection, pour some of the bubbled cutting solution into the petri dish.

**CRITICAL:** Be precise in mounting, the angle of the brain cannot be adjusted once the tissue contacts the superglue.

***Note:*** If a slicing orientation other than coronal is desired, mount brain onto vibratome chuck according to desired orientation for slicing.

4. Place chuck into the vibratome basin to begin slicing
  a. Pour remaining cutting solution from iced beaker into basin; move 95% O_2_/5% CO_2_ stone to vibratome basin as well.
  b. Program vibratome cutting window for front and back of brain, set slice thickness to 400 μm, and speed to 0.60 mm/s.
  c. Start vibratome slicing.
    ***Note:*** As each slice is cut, gently blow solution at the slice on the blade with the cut Pasteur pipette to create even slices and prevent the slice from flipping over and not completely separating when the blade reaches the forward edge of the tissue.
    ***Note:*** Slices from tissue anterior to the region of interest can be discarded into the basin for removal during cleanup.
  d. Once the hippocampus becomes visible, sections are collected into the Pasteur pipette (Figure 2D) and transferred to the mesh container in the water bath (Figure 2E).
    **CRITICAL:** Try to minimize solution exchange between the cutting basin and the slice holding container when depositing brain slices. Warm, sodium-containing ACSF will negatively impact the protective effects of the cutting solution.
  e. This is repeated until the posterior end of the hippocampus is reached and the hemispheres start to separate.
    ***Note:*** Usually, 4-5 good slices containing hippocampus of typical structure can be generated due to extent of hippocampus and sectioning thickness. Occasionally, 6 slices can be obtained.
5. Let brain slices incubate in the 95% O_2_/5% CO_2_ bubbled ACSF at 37°C for 30 min (Figure 2E).

### Microscope preparation and image acquisition protocol configuration

**Timing: 30 min**

This step takes place during the 30-minute incubation in step 5, covering both microscope powerup and system settings loading. Initial acquisition parameter creation steps will only need to be completed once. After that, reloading previous experiments allows to reuse saved settings and free up the remaining time for other activities, such as tool washing and cleanup from brain slicing.

***Note:*** This process will need to be modified depending on the type of microscope and software employed. Below is an example of how we set up our system.

6. Turn on two-photon microscope according to system start-up procedures.

**CRITICAL:** 2-photon microscopy systems are based on class IV pulsed infrared lasers. Always follow all laser safety requirements of your institution for safe laser operation.

7. Proceed to Step 8 if running the experiment the first time. If not, reuse settings from a previous run, and wait for slices to finish incubating before proceeding with Step 10.
8. Set up parameters to perform a time series experiment with z-stack using a 40X objective.
  a. Establish the Z-stack to have 60 slices with 1-micron intervals.
  b. Set the time series to have 61 cycles with an interval of 120 seconds.
    **CRITICAL:** This combination of 60 slices and 120-second intervals was chosen based on the largest number of Z-planes that were able to be completed before the 120-second interval was finished, and with 120 seconds, enough time captures occurred to be able to track changes across the time series. If 60 slices cannot be completed within that time allotment, decrease slice number or increase imaging time interval. This creates reliability in being able to compare runs.
  c. Set the frame size to 512 x 512 pixels, the imaging speed to maximum, which translates to a pixel dwell time on 1.27 μs, and the line average 2.
  d. Ensure that channels for each component being imaged are enabled and present, with the gain adjusted for each component (this will be further adjusted with the first brain slice on the microscope and then reused in all future experiments) (Troubleshooting Problem 5).
    ***Note:*** For our setup, there are 3 channels necessary. Green for the GFP-labeled microglia, Red for the pHrodo Red labeled iC3b-opsonized beads, and Blue for the Cascade Blue-labeled Dextran. All three fluorophores are excited simultaneously with light at 850nm wavelength and recorded simultaneously with three non-descanned detectors.
9. Set up parameters to take a 2.5X objective overview photo of the brain.
  a. Remove both the Z-stack and Time Series settings.
  b. Change the line averaging to 16.
  c. Adjust any laser power gain for the adjusted objective change.

### Preparing brain slices and micropipette injection solution for imaging

**Timing: 2.5 h**

This step prepares drug treatment incubation for the tissue slices and the solution that will be injected into the slice during imaging. This section concludes with brain slices being placed onto the stage for imaging.

10. Fill 2-photon microscope tissue chamber with ACSF and bubble with 95% O_2_/5% CO_2_ for the remainder of imaging
  a. Ensure even flow rate to the stage reservoir with a rate of approx. 2ml/min.
  b. Ensure placement of the vacuum outflow creates a steady fluid level in the chamber without being too low to dry out the tissue or too high to flood the stage.

**CRITICAL:** Too fast or slow flow rate will result in drifting of tissue during imaging and disrupted fluorescence intensity across 2-hour timelapse.

11. Slice container is removed from 37 °C bath and placed at 20–25°C; bubbling with 95% O_2_/5% CO_2_ persists.
  a. For drug incubation, 10 mL of ACSF with either vehicle or drug added are bubbled in containers separate from the main tissue slice container (Figure 3A).
    **CRITICAL:** These drug containers contain their own mesh system to hold the tissue. This mesh system acts as a barrier between the source of bubbled gas and the tissue is still required so the tissue is not damaged from direct gas output.

**Figure 3:**
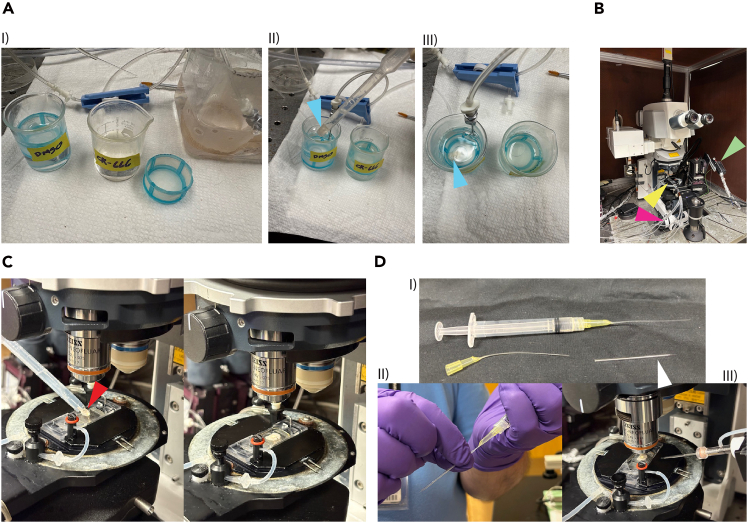
Preparation of brain slices and micropipette for experiments (A) Demonstration of CK-666 and DMSO vehicle control-containing ACSF (i), with gas exchange bubbling and slice transfer (ii, iii) (slice indicated with blue arrowhead). (B) Two-photon microscope set up demonstrating stage (yellow arrowhead), perfusion tubing (pink arrowhead), and micromanipulator for micropipette (green arrowhead). (C) Using the Pasteur pipet as during vibratome sectioning, adding the slice into the bath on the two-photon stage (red arrowhead). (D) Creating loading tips (yellow tip heads) and loading micropipettes (white arrowhead) (i), loading the bead mixture into the micropipette (ii), and loading the micropipette into the micromanipulator before positioning it near the tissue slice (iii).
  b. One tissue slice is transferred from the main tissue container to the drug or vehicle container and bubbled for 2 h (Figure 3A).
    **Pause point:** If no non-treated tissue data is required, then this is a stopping point for the 2-hour incubation period required to inoculate the tissue slice with drug or vehicle control. If a non-treatment tissue run is being performed (recommended), then move the first treated tissue into inoculation and continue now with steps for the non-treated slice.
  c. Using the transfer Pasteur pipette as before, move one tissue slice to the tissue chamber on the 2-photon microscope stage (Figures 3B and 3C).
  d. Place the slice anchor over the tissue to secure it in place for imaging (shown in Figure 3C, right).
12. Prepare the injection solution to a total of 250 μL with the following components:

**Table:** 

Solution Component	Amount (in μL)
Cascade Blue labelled 3000MW Dextran (10mg/mL)	15
iC3b-pHrodo 2 μm beads	1.5
DMSO or CK-666 200μM (optional)	0.8
ACSF	233.5 (or 232.7)
**Total**	**250 μL**

**CRITICAL:** Ensure that all components are centrifuged and only draw from supernatants to prevent any particles from blocking the micropipette. Ensure that ACSF is filtered. Clear solutions by eye are not necessarily clean enough. The exception is the beads, which need to be resuspended in order to be transferred to the injection solution.

13. Using a 3 mL syringe and a pipette tip, take up the entire solution made in Step 12.
14. Take a lighter and a P200 tip, heat the tip until slightly melted so it can be pulled to create a very fine filament.
  a. Cut the filament with a razor about 7.5 cm (3 inches) below the loading end of the tip.
  b. Mount the newly crafted p200 tip onto the filled 3 mL syringe, resembling Figure 3D(i).
  c. Holding the syringe in one hand and the micropipette in the other, fill the micropipette roughly 4 cm of the way full, making sure to minimize bubbles (Figure 3D(ii)).
    ***Note:*** The first time this is done, the filament might not pull correctly and may be blocked. Multiple tips may be required to successfully fill the micropipette. Additionally, for each change of pipette solution, a new tip should be used to avoid cross contamination of stimuli.
    While we prefer fabrication of single use loading tips from pipette tips for one time use (minimizing contamination) and ready availability, pipettes can also be loaded with commercially available flexible needles like MicroFil ™ from WPI.
  d. Tap and shake the micropipette to dislodge any remaining bubbles in the tip.
  e. Load the micropipette onto the micromanipulator (Figure 3D(iii)).

### Choosing a position in the brain slice and running the acquisition

**Timing: 2.5 h**

This section involves choosing the area of the brain slice that will be imaged, ensuring the micropipette is clear and can inject into the tissue, placing the micropipette into the tissue, and starting the run.

15. Select the area of the brain slice to image and prepare the objective.
  a. Using the 2.5x objective, focus on an area of the CA1 between two harp threads.
  b. Switch the objective to the 40x and focus until the surface of the cortex comes into focus.
  c. Move inwards on the tissue towards the hippocampus until the pyramidal cell layer of the CA1 is visible.
    ***Note:*** Our study involved recordings in the hippocampus and finding the area is described here. Any brain region of interest within the slice can be targeted and working with a clearly identifiable landmark and a consistent strategy to move to the desired location will be necessary.
  d. Raise the objective a few hundred micrometers to be able to bring in the micropipette. Do not move the stage.
16. Move the micropipette underneath the objective, going back and forth, and advance towards the focal plane, until the shadow of the micropipette is visible under the objective. If it is not, see Troubleshooting Problem 6.
  a. Once the shadow is seen, the micropipette can be brought into focus (Figure 4A, left).

**Figure 4:**
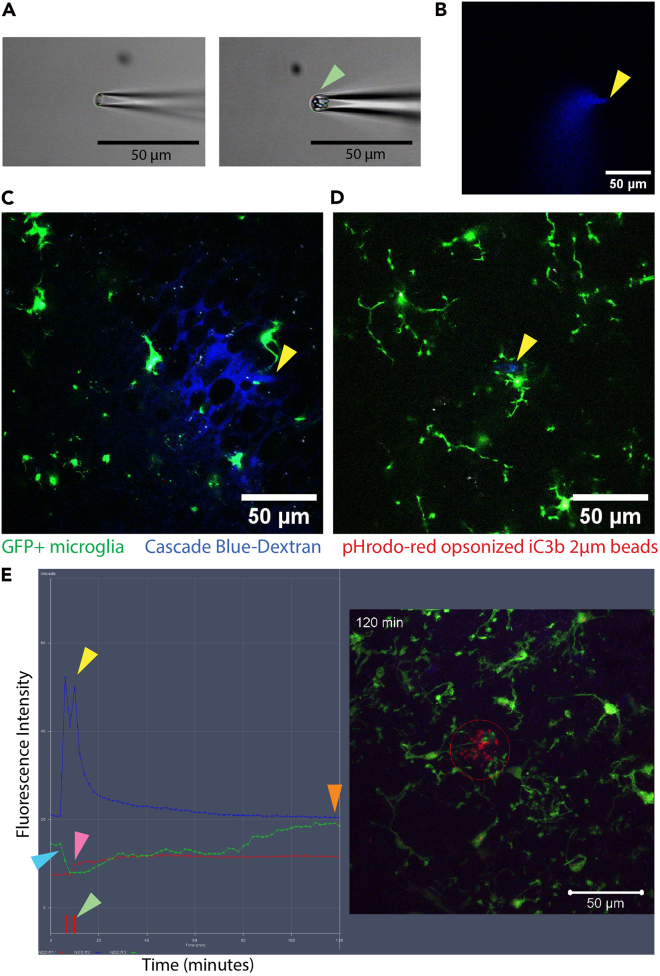
Preparing the micropipette before injection (A) The presence of beads is verified by making a puff above the tissue (green arrowhead). (B) The dextran cloud is visualized to determine flow rate, also above the tissue (yellow arrowhead marking the end of the micropipette). (C) Micropipette is lowered until it touches the tissue surface (yellow arrowhead marking end of tip). (D) Image of tissue at a depth of 50 μm below the surface, where only one section of the pipette is visible (yellow arrowhead). (E) Example time course from Zeiss software over the Region of Interest (ROI) placed onto the image. The red circle (right) displays where the ROI is located. The time graph on the left shows the changes in all three channel fluorescence intensities within that ROI over the 2 h. The yellow arrowhead shows the peaking of dextran intensity during the initial injection(s). The blue arrowhead shows that the microglial process present right around the end of the micropipette was briefly blown away at the time of injection (seen through the drop in green intensity). The pink arrowhead demonstrates when the red beads were deposited into the tissue. The green arrowhead shows injection times. In this case, there were 3 injections needed to deposit the correct number of beads into the tissue. The orange arrowhead is looking at the endpoint fluorescence intensity of the three channels. The blue line for the dextran has returned to its baseline value before injection. The red line has stayed the same since injection, pointing towards the beads staying in focus and not drifting. The green line has steadily increased until reaching this timepoint, alluding to process interaction with beads and increased microglial process presence in the ROI.
  b. Using the objective focus, look up and down the length of the micropipette to ensure that there are beads present in the length of the pipette and that there are no debris or bubbles present (Troubleshooting Problem 4).
17. Perform a test puff to ensure that the beads flow through the tip.
  a. Place the objective focus at the end of the micropipette (Figure 4A, left).
  b. Puff at 4 pounds per square inch (PSI) for 5 seconds using the pico-spritzer.
  c. Beads need to be physically seen come out past the tip end to ensure that everything is flowing well (Figure 4A, right (green arrow depicting beads)).

**CRITICAL:** If beads are not seen flowing past, multiple puffs may be needed until beads are released. If debris causes a blockage during these puffs, then higher pressure puffs may need to occur to drive out the blockage (troubleshooting problem 4).

18. Bring micropipette down closer towards the tissue and switch to fluorescence illumination.
  a. Ensure the color for the dextran (e.g., blue) is the channel currently active.
19. Go Live on the Zeiss software.
  a. Adjust Z focus until micropipette comes into focus (Figure 4B).
  b. Adjust the baseline pressure of the pico-spritzer (balance) on the micropipette so that a gentle constant flow is occurring.

***Note:*** Gentle constant flow from the micropipette is needed to avoid debris from the bath being sucked into the pipette and clogging it. It also allows us to visualize the end of the pipette that otherwise would not be visible. Pressure should eject a minimum amount of solution to not deposit it on the tissue surface or eject it into the tissue before additional pressure is applied but enough to avoid flow into the pipette through capillary force. The baseline pressure (balance) of the pico-spritzer to achieve this varies depending on minute changes in micropipette tip geometry but the balance will usually range between 0.1 and 0.4 PSI.

20. Bring the micropipette down to touch the tissue (Figure 4C).

***Note:*** It will be obvious when the micropipette meets the tissue, as the cloud of dextran will decrease, and there will just be a blue outline of the micropipette filled with dextran that is now blocked up due to the tissue presence.

21. On the Z-axis measurement for the Z-stack, zero out the Z-axis, so it is counting the top of the tissue as the 0 point.
  a. Switch the micromanipulator into the 4^th^ axis mode (matching the angle of the micropipette) and begin advancing into the tissue.
    ***Note:*** On our setup we used an angle of 25 degrees, but anything between 20 and 35 degrees works well. The ultimate number depends on the geometry of the setup (height of stage, location of micromanipulator, length of pipette holder).
    **CRITICAL:** Ensure that the micromanipulator is set to slow speed mode, so the micropipette doesn’t tear through the tissue.
  b. Move both micropipette and focus in live view mode of cells over and down on the z-axis to keep the micropipette tip in focus while moving deeper into the tissue.
    ***Note:*** If the micropipette becomes dim or hard to discern when moving, please see troubleshooting problem 7.
22. Once the micropipette tip reaches −60 μm, begin looking around for cells nearby (Figure 4D). Within the next 20 μm, find a good spot to place the tip of the micropipette.

***Note:*** A good spot is defined as a small area, roughly 20 μm, that does not have cell process endings either in the plane of the pipette or in a few microns either above or below. This will allow space to inject the beads without placing them directly on top of cells, allowing us to watch cells react to the stimuli and respond in a way that is trackable (looking at quantitative data such as process length and speed of response). Placing the beads too close to cells may cause differences in responses slice to slice as the cells have to respond more or less for an interaction. If the micropipette tip ends up on top of a microglial cell, push forward another few microns to go past the cell where there is potentially an open space, as in Figure 4D.

23. Center the Z-stack 10 μm above the Z plane where the tip of the micropipette is located.

***Note:*** Centering is done above the micropipette tip as the beads tend to drift “up” in the tissue, thereby placing the majority of the stack above the micropipette tip allows for capturing the most cell-bead interactions

24. Start experimental run on the imaging software.
25. Wait for 3 cycles to pass to establish baseline readings of the cells.
26. Inject the beads into the tissue.
  a. Activate Pico spritzer for a 5 second injection at 4 psi.
    **CRITICAL:** This value may need to be troubleshot depending on differences in injection equipment and pipette geometry between lab setups.
    ***Note:*** For other applications, this value changes. For example, the chemotaxis experiments used 4 psi for 3 seconds.
  b. Watch the next cycle to ensure that at least 12 beads are deposited into the tissue (Troubleshooting Problem 8).
  c. Draw a region of interest circle around the beads and open the region of interest fluorescence intensity vs time graph (Figure 4E).
    **CRITICAL:** The region of interest intensity graph can be used to determine if the dextran is decreasing as it dissipates into the tissue, or whether it is still flowing out of the micropipette (troubleshooting problem 16), and watch process interaction with bead fluorescence, as well as track bead movement into/out of the region of interest to detect any drift in tissue (troubleshooting problem 11).
    ***Note:*** A region of interest circle can also be drawn around the tip of the micropipette during a chemotaxis assay for a similar purpose.
27. Run has started and will continue for 2 h.

**CRITICAL:** For other issues that may arise post-injection or during the run, please see troubleshooting problems 9-16.

### Taking the final image of the bead interaction run

**Timing: 10 min**

This section outlines changing the settings in the software to take a higher resolution final image for the run, to better examine the interaction between the cells and the beads at the 2-hour timepoint, as well as capturing the plume location of the injection.

28. Once run has finished, save run file.
  a. Create a maximum intensity projection of the just acquired image within the Zen software to judge the quality and success of the run.
  b. Save maximum intensity image as well (example frames from time series seen in Figure 5A).

**Figure 5:**
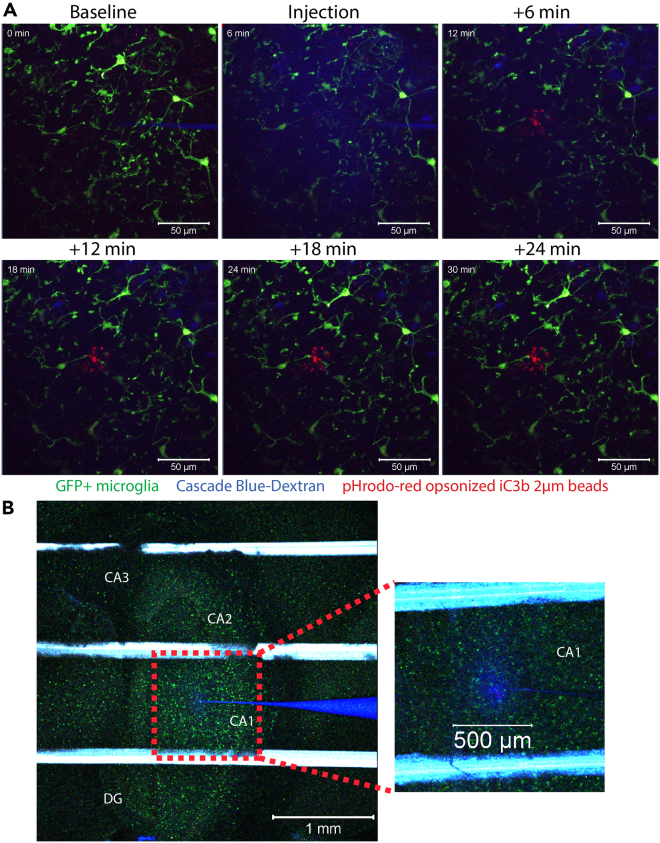
Example data output (A) Representative images from the first 30 min of a bead interaction acquisition run demonstrated in Figure 6E. Baseline can be seen in the first panel (time = 0 min); there are no red beads present. The blue dextran in the pipette is also evident in these maximum intensity projection images. The second panel shows the injection, as there is a blue dextran presence throughout the image. The following four panels show timepoints at 6-minute intervals after injection, demonstrating the blue dispersing, the beads appearing and settling, and the green microglial processes starting to come in and interact with the beads. Images were taken every 2 min, but every 6 min are shown here. Scale bars represent 50 μm. (B) Example image from 2.5X objective size, demonstrating where exactly this specific injection occurred, compared to anatomical landmarks for parts of the hippocampus, such as the CA1 region, CA2, CA3, and dentate gyrus (DG). Subset (right) shows the end of the micropipette in clearer detail. Scale bars represent 1 mm (left) and 500 μm (right subset).
29. Go back to stack settings to acquire a new image.
  a. Change averaging to 8x.
  b. If stack has slightly drifted, center of Z plane can be moved, or expanded (if more than 60 μm is required to encompass all bead interactions).
  c. Remember to turn off timelapse (uncheck box).
30. Start Experiment.
31. When finished (should be roughly 8 min), save file.
  a. Prepare and save a maximum intensity image.
32. Change objective to 2.5x to document the location and extent of the injection site (the dextran plume will be visible at 2.5x).
  a. Reuse settings from the 2.5x objective settings established earlier.
  b. Adjust focus until the brain tissue is visible.
  c. Push “snap” to take a single overview image.
  d. Save file (example seen in Figure 5B).

### Repeating with the second brain tissue slice

**Timing: 2.5 h**

This section outlines how to repeat the process and can be done for multiple image videos with slices. This step is repeatable as many times as there is time for runs or tissue available.

***Note:*** Steps 33–35 can be skipped if imaging is being performed on the other hemisphere of the current slice of tissue (recommended for short imaging runs, such as 1 hour, not recommended for multiple drug treatments with long imaging runs, such as 2 h).

33. Raise microscope objective and remove harp to remove the current tissue from the chamber.
  ***Note:*** Tissue can be dislodged from the supporting threads by puffing at it with the cut Pasteur pipette until it lifts and is floating in the bath solution.
  a. Tissue can be discarded once imaging is done.
34. Place a new tissue slice on the microscope.
  a. This slice was most likely taken from either drug or vehicle incubations, whichever one was established earlier.
35. Place a different (new) tissue slice into an incubation container.

**CRITICAL:** The last time through this section of the protocol, step 35 is skipped.

36. Load a new micropipette with a new solution.
  a. A new mixture will need to be made, adding in the vehicle or drug matching the condition from the pre-incubation this new brain slice came from.

**CRITICAL:** Resuspend bead mixture again before using; if other solutions have gotten mixed up, another spin down is recommended.

37. Repeat micropipette testing to ensure there is no blockage and beads are dispersed.
38. Repeat imaging preparation (finding where on the tissue to image, bringing the micropipette down to the tissue, etc.) and performing the 2-hour run.
39. Repeat final imaging settings for a higher-quality final image, and repeat the 2.5x image for brain hemisphere overview imaging.
40. Repeat steps 33–39 as needed until all imaging has been completed.

## Expected outcomes

Overall, successful completion of this protocol is expected to generate high-quality imaging data that will lead to quantitative insight into how microglia interact with phagocytic targets *in situ*. The main imaging output is a time series captured across three imaging channels of dextran (blue), GFP (green), and beads (red). The beads should disperse into the tissue along with the dextran, and GFP-expressing microglia should begin interacting via their processes with the beads. Please see Figure 5A for example images. The image series will also contain associated imaging metadata that will aid reproducibility and downstream quantification. Individual frames can be isolated, or the entire series can be presented as a timelapse video.

The expected quantitative outcomes can be quite broad and depend on the scope of the experiment. First, an existing morphological analysis pipeline (such as the MotiQ software)^14^ can be used to generate information on microglial structure before and after bead introduction. Second, the physical interaction of microglia with beads can be assessed quantitatively over time through counting of process interaction with beads. Finally, the dynamic nature of microglial processes generated in response to phagocytic targets (including their persistence, distance travelled, and velocity) can easily be quantified from timelapse data.

**Figure 6:**
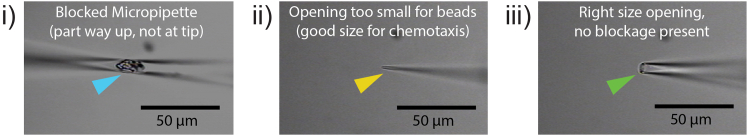
Troubleshooting – examples of reasons the micropipette is not dispensing beads (i) Representative image of a clump of beads and other debris that has blocked the micropipette further up the length away from the tip. (ii) Representative image of a clear micropipette with a small opening, resulting in lack of bead dispersion. This size opening would be beneficial for injections that do not require physical particle deposition, such as chemotaxis. (iii) Representative image of a correctly sized micropipette clear of debris or blockage, ready for injection of beads. Scale bars represent 50 μm.

We have utilized this experimental protocol successfully in a recent publication: Paulson et al., *EMBO Reports* (2026).^1^ Figures 4, 5, and 6 of that publication contain images and analysis generated using this workflow. There are also additional movies and data in the supplemental material associated with that publication.

## Quantification and statistical analysis

There are multiple avenues of analysis that can be performed using the output from these videos. Examples include endpoint analysis of bead interaction percentage, process motility dynamics, and overall cell morphology changes.

For the endpoint analysis of bead interaction, the software analysis program Arivis 4D was used.^15^ This program allowed for the creation of a 3D projection of the end point image stack taken. This 3D projection was used to manually count the number of beads with some green overlap compared to the total number of beads present in the field of view. Creating the 3D projection of the end point image stack and enabling rotation of the field of view to ensure that overlapping beads are near a process and not in a different Z-axis position prevents false positive conclusions. For our research, this was performed only at endpoint, but this could easily be done in a stepwise fashion over time to look at increase in interaction between the beads and the cells over time.

For process motility dynamics, the manual tracking plugin pre-loaded on the FIJI version of ImageJ^16^ was utilized from the start of injection to the video end. Each track was saved and compiled until all processes in the field of view near a bead were tracked (a maximum of 15 processes per field of view). Using the Ibidi chemotaxis plugin^17^ to analyze the raw data, outputs such as process velocity, persistence, and distance travelled could be measured. Persistence is a ratio of the direct line between the starting and ending position (d) and the total path length traveled during the same period (T), so d/T (persistence) is a value from 0 to 1, with larger values meaning higher persistence. Since this is done manually, tracks could be stopped after the process interacted with its first bead, rather than forced to do the entire video as with automated tracking. By stopping at the first bead, process variation in how long they spend at the first bead before moving to the second would not skew track persistence and velocity outputs measured. These outputs allow for examination of the cell response to opsonin signal and can be averaged and compared across treatment groups. The length of the process was also measured using the measure tool on ImageJ to determine the distance from the cell body to the process end, independent of bead interaction, at the endpoint of the video, demonstrating the locality of the signal-cell interaction.

For other applications of this process, such as adding ATP for a chemotactic measurement, the measure tool on ImageJ can be used to determine the length of the visible micropipette and other factors. Manual tracking was also similarly used on these processes, measuring from the injection timepoint to when the process reached the tip of the micropipette.

Lastly, cell images can be fed into the analysis software called MotiQ.^14^ For morphological changes over time comparisons, such as found in Paulson et al, EMBO Reports (2026),^1^ we altered the imaging parameters slightly, imaging cells with a line average of 4 and pixel size of 0.42 μm over a shorter time window (only 60 second intervals), resulting in 10-minute videos with 1-minute intervals at a higher definition of the cell. The MotiQ plugin produces cell skeletonizations, and process dynamics such as extension and retraction over time, quantification of branching dynamics of the cells such as branch points and number of process tips, and quantification of the cell body area compared to total cell area.

We used one-way ANOVA or t-tests depending on the number of comparison groups. ANOVAs were mostly used if untreated samples were compared to both vehicle and inhibitor treatments.

Exclusion criteria were established to correct problems with the mice or health of slices. Exclusion pertained to scratching an entire run or an entire mouse prior to initiating analysis and never involved excluding individual cells while running analysis. Examining cell morphology and cell death during untreated and vehicle-treated bead interaction runs ensured that the cells that were too stressed to respond to any stimulus were not included and therefore did not skew results during analysis. During runs that were measured, all cells proximal to a bead injection had their processes measured. If more processes were present and interacting than the 15-process cap, then processes were selected at random throughout the field of view, not favoring one cell or cluster over others in order to gain a full representative view of the microglial response under those conditions.

## Limitations

There are several possible limitations to this protocol, mainly related to issues with tissue health and the generation of unusable data. Slice generation can be a challenge, as there are multiple points where the protocol may be compromised. Extracting the brain without damaging is important as knicks or cuts in the brain cortex can cause posterior slices to prematurely fall apart and prevent reliable imaging. Not having a straight cut when separating the cerebellum from the rest of the brain or mounting the brain onto the vibratome chuck at an angle may prevent the highest usable number of slices from a brain due to uneven slicing or the hippocampus not being even across the slice. In addition, sectioning of the tissue must be quickly processed and moved to recovery ACSF to limit cell death, minimizing oxygen deprivation and preventing metabolic rundown. These steps may require some practice. Each of these steps may result in the mouse tissue being suboptimal for imaging.

In addition, the mouse brain tissue may not sustain sitting long-term due to issues resulting during slice preparation, thus causing bulging and imaging disparities. Posterior tissue slices may start to fall apart when sitting long-term in the ACSF beakers, if saved for the end of the day to be placed on the microscope. This can lead to issues with tissue drifting, focus drift, and image analysis of the resulting run video. In runs such as these, it is important to have parameters in place to determine if the cells are still viable enough to analyze. On average, 3-5 good slices were obtained per mouse brain containing the ideal area of the hippocampus desired for these experiments. Running each slice was roughly 2.5 hour, pointing towards the last run finishing 8–10 h after the slices came off the vibratome. We found that the limitation wasnot the health of the slices but rather the length of time spent imaging and the lack of more than 3-5 viable slices per day.

While parameters are in place to ensure that the injection occurs in the same place each time (80 μm below the tissue surface, below the pyramidal cell layer of the CA1 region of the hippocampus, in the stratum radiatum), some of that placement depends on practice and expertise using the 2-photon microscope and familiarity with the angle and depth of micropipette placement via the micromanipulator. Moving even slightly closer or further away from the neuronal cell body layer may result in changes in microglia number. An ideal location maintained nearby processes from at least 4–6 microglial cells, whether at the plane of the micropipette or just above or below it, in order to determine an average cellular response to stimuli. In addition, mouse to mouse differences may result in more or fewer microglia present in the same injection location. Once the micropipette has been inserted, it cannot be pulled out and reinserted on that same tissue side, due to too much disturbance of the tissue, so a decision must be made immediately about moving over to the other hemisphere of the tissue. Having too few microglia present can skew analysis results dependent on the number of cells interacting with a cue, and other analysis dependent on cell signaling.

Finally, some tissue may not behave as expected. Exclusion criteria with non-treatment runs can ensure that brain tissue with cells that do not respond as expected can be excluded as an entire mouse tissue set (i.e., cell death, preservation of slices through imaging, etc.). There may be no identifiable reason why the tissue is not reacting properly (i.e., the mouse was euthanized well, the brain collected quickly and sliced well, and there were no issues with mounting), but nonetheless the entire brain tissue should be excluded from experimentation and analysis.

## Troubleshooting

### Problem 1

Beads are not labeled with pHrodo (before you begin Step 3).

### Potential solution

Different opsonins require slightly different bead incubation to ensure bead labelling. For example, in our hands, IgG labeled beads require a 2-hour label at 37°C followed by 3 washes rather than the 16–18 h at 4°C that the iC3b-beads require. Troubleshooting bead labelling and opsonization should ideally be completed with *in vitro* cells rather than brain tissue, if possible, to minimize unnecessary use of mouse tissue.

### Problem 2

Beads are over-labeled with pHrodo (resuspended bead mixture is pink, not milky white, after washes) (before you begin Step 7).

### Potential solution

Incubating the beads too long during the 16–18 hour shake (i.e., 24–36 h) can result in residual dye labeling the beads and creating a false sense of opsonin label amount. It is important to ensure adherence to the amount of time required for bead labeling on the rocker. The way to determine here if they are over-labeled with pHrodo dye is to determine if the beads are brighter than normal at the normal red fluorescence setting established for this experiment.

### Problem 3

Superglue doesn’t hold when placing the mounted brain into the cutting solution on the vibratome (Step 2b).

### Potential solution

The superglue was too dry before mounting the brain, and the brain was unable to adhere. While the brain is mounted more reliably when the superglue is not too runny, if it becomes too tacky, the tissue will not bind solidly to the chuck. Wait slightly less time to mount tissue (try 30 seconds).

### Problem 4

Micropipettes are blocked and therefore unable to dispense beads (Figure 6I, black arrowhead). This can also apply when bubbles are present or when there is no blockage visible, but no fluid is ejected (meaning that the blockage, either by debris or bubbles, is higher up in the micropipette) (step 16b).

### Potential solution

To prevent blockage to the best ability, ensure that the ACSF solution is filtered before usage. Chemical crystalline compounds may appear dissolved, but are not, and will block the micropipette. In addition, all other components that are made up should be centrifuged to settle any debris, and only the supernatant should be taken (i.e., for the dextran, drugs) to ensure that the additives are clear as well. In addition, after pulling micropipettes, store them in a way to prevent lint or other dust and debris from falling down the shaft of the micropipette and blocking it when loaded with solution.

If there is debris in the shaft when examining the micropipette, a strong puff with increased psi is the first solution to be able to clear out the tip and push the debris out the end. If this does not work, attaching a large syringe to the line and instead sucking up the line could move the debris in the other direction, further up the micropipette. If this course is taken, another test push is necessary to ensure that the blockage does not come back down or that it is not blocking the micropipette further up the shaft.

A test push to watch beads flow out after clearing any debris is necessary before taking the micropipette down to the tissue for injection. Seeing beads flow is necessary to ensure that they are nearby and will be injected during the puff.

Ultimately, a fresh micropipette should be used if no reliable bead ejection can be verified. If the problem appears to originate from the solution, it is time to make up a new 250 μL solution or re-centrifuge and filter the solution components.

### Problem 5

Cells are too bright when establishing laser settings (Step 8 or Step 20).

### Potential solution

We bred our CX3CR1^GFP/GFP^ mice to a C57BL/6 mouse and used the F1 mice (CX3CR1^GFP/+^) aged appropriately for our study. We found the heterozygous GFP expression was still very bright and easily viewable. The optimal excitation wavelength for the dextran and the red beads is 800 nm, while GFP is optimally excited at 950 nm. We used 850 nm as the excitation wavelength to acquire all three channels at the same time. If the laser power needed to visualize the dextran overexposes the GFP, then lowering the detector gain as much as needed will help to avoid saturated areas in the microglia.

### Problem 6

The micropipette starts to angle when moving, digging into the tissue. Alternatively, the micropipette shadow is not detectable under the objective when trying to locate it above the tissue. (Step 16 or Step 21b).

### Potential solution

If the micropipette shaft is too short or not tapered enough, then the micropipette will push into the objective before one is able to get the micropipette into the correct position for the injection. In addition, as the objective lowers to the tissue, the micropipette will be pushed into the tissue, causing it to rip through the tissue, damaging cells, and not allowing for reliable placement. The only solution for this is to choose a longer micropipette. Ensure that, when pulling the micropipettes and breaking them to create the correct size opening, not to break it too short.

### Problem 7

**Figure 7:**
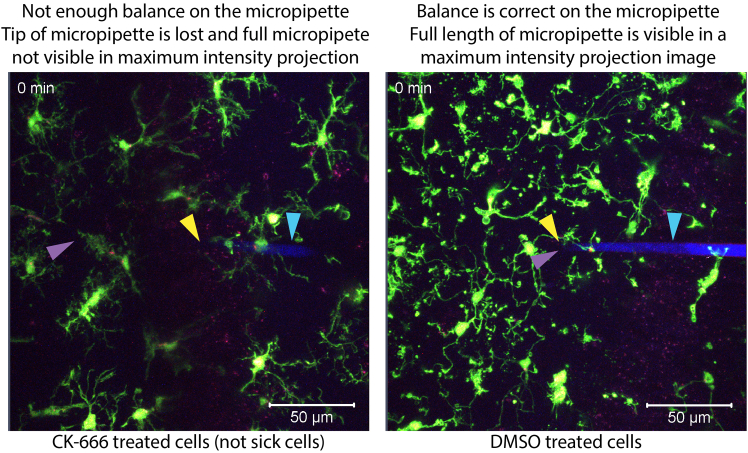
Troubleshooting – examples of micropipette flow set incorrectly, resulting in loss of visualization Incorrect balance pressure, such as too little (preventing just oozing beads and drug out of the micropipette), can cause an inflow from the bath liquid and tissue ECM, causing a loss of visualization of the micropipette tip (left image). The blue arrowhead in both images points out the visible part of the micropipette that is present in a maximum intensity projection of the imaging stack. The yellow arrowhead demonstrates where we thought the tip of the micropipette was when we started the run. The purple arrowhead demonstrates where the tip actually was. With a loss of visualization, it is easy to wrongly center the z-stack in the tissue, resulting in the injection not being in frame and wasting the current image acquisition run. Scale bars represent 50 μm.

Flow through the micropipette is not occurring, making it very hard to discern the micropipette location in the tissue (Figure 7). This can result in losing awareness of the pipette’s position, resulting in the micropipette ending up too deep or shallow in tissue or out of frame when the acquisition starts. This makes the run inviable (Step 21b).

### Potential solution

Capillary force will suck fluid from the bath into the micropipette when immersed in the bath. To counter this effect and avoid clogging the micropipette with debris from the bath as well as losing visibility of the dextran in the tip, a small constant positive pressure (balance) is applied. Balance flow pressure to the micropipette is adjusted when the micropipette is above the tissue. This ensures that the balance outflow is not too high (spurting out dextran and beads) or too low (fluid gets forced into the micropipette). It is important to have the base flow high enough that when the micropipette comes in contact with the tissue, and the outflow is diminished, the tip is filled to the end with blue dextran. If the micropipette becomes invisible, either the balance pressure should be raised (we routinely used between 0.2 and 0.4 psi), or more dextran should be added to the solution to increase visibility.

### Problem 8

Very few, if any, beads are present after the injection within the tissue, despite the test push above the tissue demonstrating beads flowing through the micropipette (proving there are beads present) (Step 26b).

### Potential solution

There are a few different reasons that this might have happened.

• This might be due to too much time passing between making the solution and performing the injection. This causes the beads to settle within either the syringe itself or the micropipette.
• If the beads were not mixed properly into the final solution, most of the beads may be either at the top or bottom of the solution within the 1.5 mL Eppendorf tube, meaning either they are still in the syringe or most of what was in the micropipette came out during the test pushes in the beginning
• The beads are sticky due to not enough washing and/or resuspension issues (the smaller the amount of liquid they are being resuspended in, the harder it will be to break them apart), and are in large clumps, and one of these clumps is stuck higher up in the micropipette
• An air bubble or other debris is blocking higher up in the micropipette that did not come down until after doing several test puffs, resulting in the beads that had been in front of the bubble/debris being expelled in the test pushes, but now there is none left to expel.

To solve this problem, start by giving a larger push, usually in force but also can be in length, to try to drive more beads out. Increases can be up to 8–16 psi for up to 10 seconds. This is usually done within 1-2 imaging cycles of the initial push, once it is noticeable that there are no/not enough beads present in the image frame for analysis. This will either place a lot of beads now into the field of view, or just a lot of dextran. The recommended number of beads present should be more than 12. If only a few or no beads are added, one more large push can be done (for a total of three pushes), and then that hemisphere of tissue should be discarded. Pull out the micropipette, replace it with a freshly filled one, and try again on the other side of the tissue. When trying again, it is recommended to flick the solution in the syringe several times to try to disperse the beads better in case they are clumped at the top of the solution.

### Problem 9

**Figure 8:**
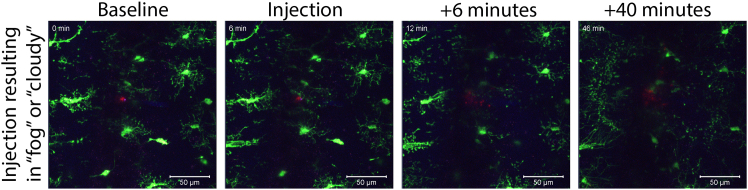
Troubleshooting – example time series of a cloudy injection After injection, the area directly around the micropipette tip becomes dim and does not recover. A cloud or “fog” is deposited with the injection, making it very difficult to correctly track processes and measure interaction with beads. Scale bars represent 50 μm.

There is consistently cloudiness in the visual field upon injection, obscuring crisp outlines of cells near the injection point (after Step 26b) (Figure 8).

### Potential solution

One of the components in the injection solution may be causing the cloudy texture. Fine pore size filters, such as those found on filter syringes, may be causing the loose granules to become almost powered and give off a cloudy appearance upon injection. We mainly found this after dissolving our ATP in ACSF, which created a cloudiness when using it when filtered through a filter syringe. While it is true that large crystals can block the injection micropipette, we found that using syringe filters may cause the solution to be hazy.

Instead, for solutions such as drug injections, centrifuging the solution for 30 seconds at 16,000 x g and then only taking from the supernatant will prevent any crystals from blocking the micropipette and still allow these drugs (or even the dextran) to be ejected into the tissue.

### Problem 10

**Figure 9:**
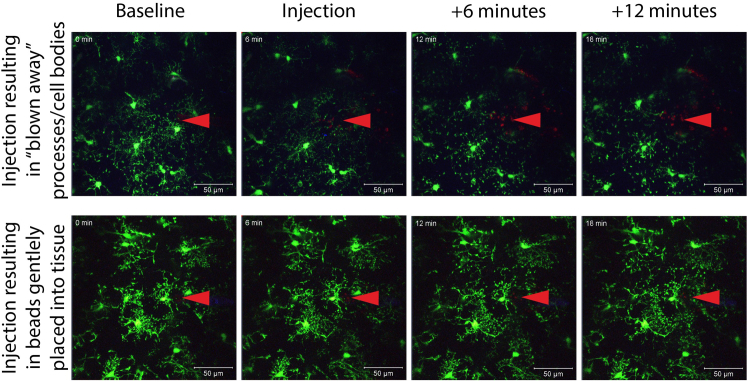
Troubleshooting – example time series of cell processes and even cell bodies being pushed away within the imaging area from a very strong injection High pressure or extended time of injection can result in “blowing away” the cells that are near the micropipette tip. The red arrowhead in both time series represents where the end of the micropipette tip is located. In the top series, where there is too much injection pressure, the processes that were near the cells are dispersed and do not recover. In addition, a couple of cell bodies in the upper half of the field of view vanish after the injection. In contrast, the bottom series demonstrates depositing beads into the tissue without disrupting or displacing processes or cell bodies of the surrounding tissue. Scale bars represent 50 μm.

After injection, cells are no longer near the micropipette tip. These cells were “blown away” following the injection (after Step 26b) (Figure 9).

### Potential solution

The pressure behind the injection, or speed of it, was too high and caused a cavity around the tip of the micropipette to be created. Lessen the pressure of injection and increase the time of injection to deposit beads around the cells without disrupting the cell microenvironment.

Another cause may be that the tip opening is too large. Try for a slightly smaller tip opening to prevent too much force being applied to the surrounding environment. The goal is to deposit beads in a nice spread without disrupting the cells and extracellular matrix of the brain too much.

### Problem 11

**Figure 10:**
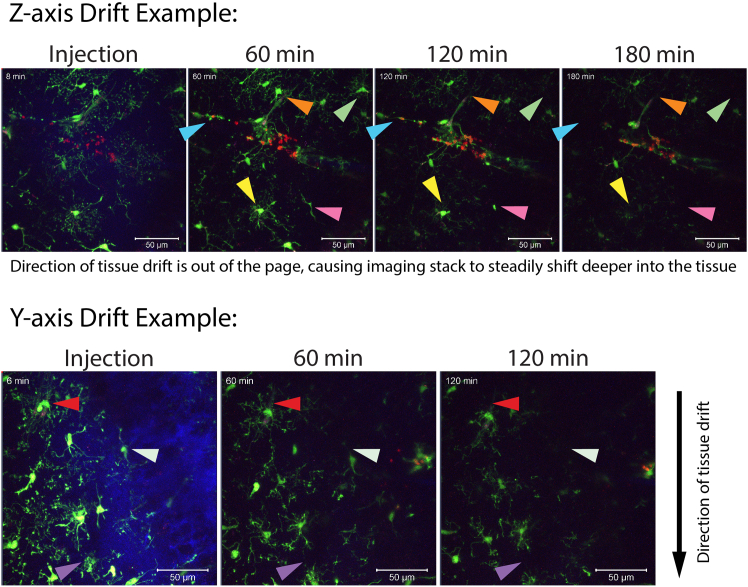
Troubleshooting – examples of drifting in either the Z-axis or Y-axis of the field of view Drifting can happen due to slow or fast bath flow or due to tissue bulging from cortex breakdown. Examples are provided for both types of drift. Arrows are placed either at the 60-minute mark (Z-axis example) or at the injection timepoint (Y-axis examples), and are not moved in subsequent timepoints, demonstrating the change in cell soma locations that drift away from arrowhead points. In the Z-axis, the tissue is drifting out of the plane of view, so the focal plane is diving deeper into the tissue, causing the loss of visibility of imaged processes. This also explains the cell somas disappearing over the course of one hour. The Y-axis drift example has the tissue drifting down within the frame, meaning the cell somas are slowly moving down compared to the arrowheads. Scale bars represent 50 μm.

Over the course of the timelapse, the video field of view drifts, making analysis of tip interaction difficult or impossible (after Step 27) (Figure 10).

### Potential solution

If the tissue is drifting along the X-Y axis, the problem is most likely the rate of the bath flow. Having the flow rate too high or too low or having the fluid level in the chamber too high or too low, can cause the tissue to move or even bulge between the strings on the harp keeping it in place. Without a strong enough flow the bath level can drop, changing the optical path and even cause the loss of solution making contact under the dipping objective. The solution to this problem is to increase flow rate of the bath solution. Caution is needed, as too much flow can lead to overflow from the chamber and damage the microscope and is wasteful. There is no advantage to excessively increasing fluid exchange speed. We found a speed of 2ml/min worked well but expect a range of 1-3ml/min to be workable.

If the tissue is drifting in the Z-axis, tissue drift could be due to tissue breakdown. The more posterior pieces of the hippocampal region do start to separate between the hippocampus and the tissue directly ventral to the hippocampus. This loss of hemisphere integrity can cause the hippocampal region to bulge in between the harp strings and result in the image drifting as the tissue is pushed into a different focal plane through bath flow and the loss of securement holding it in place. While posterior hippocampal slices can be used, care should be taken to move these pieces the fewest times possible (i.e., not to place them into a drug bath and then onto the microscope instead of directly onto the microscope) to lower the rate of hippocampal detachment. In addition, if enough anterior hippocampal regions are secured, they should be favored for data collection over posterior slices. This tissue breakdown drift may also result in the video subsequently growing dimmer over the length of the video, if the tissue is bulging and the depth of the imaging area becomes darker as it becomes a lot deeper than initially intended.

One solution for minor Z-axis drift is placing the pipette injection 10 slices below the center of the stack, so that as beads rise and as the minor Z-axis rise occurs, most of the interactions remain in frame to observe.

### Problem 12

The cells appear to be getting dimmer as the run progresses, and the cells look like they are more stressed (less process extension, less interaction with the injection site or micropipette contents) (after Step 27).

### Potential solution

If the cells are dying or becoming stressed independent of treatment groups, and the image is also potentially growing dimmer as the video goes on, the laser power may be set too high for cell health.

Exciting all fluorophores at 850nm, we routinely used a laser power of 35%. More than 50% laser power at this wavelength might begin to impact cell viability, power below 25% tended to not optimally use the dynamic range of the detectors and led to poor image quality. Cells may be dying from phototoxicity. Lower the laser power settings and repeat run on fresh tissue to see if the problem is fixed. If this is something that has started spontaneously part way through experimental days, return to a previous good day and reuse those parameters on the microscope, as something may have changed.

### Problem 13

**Figure 11:**
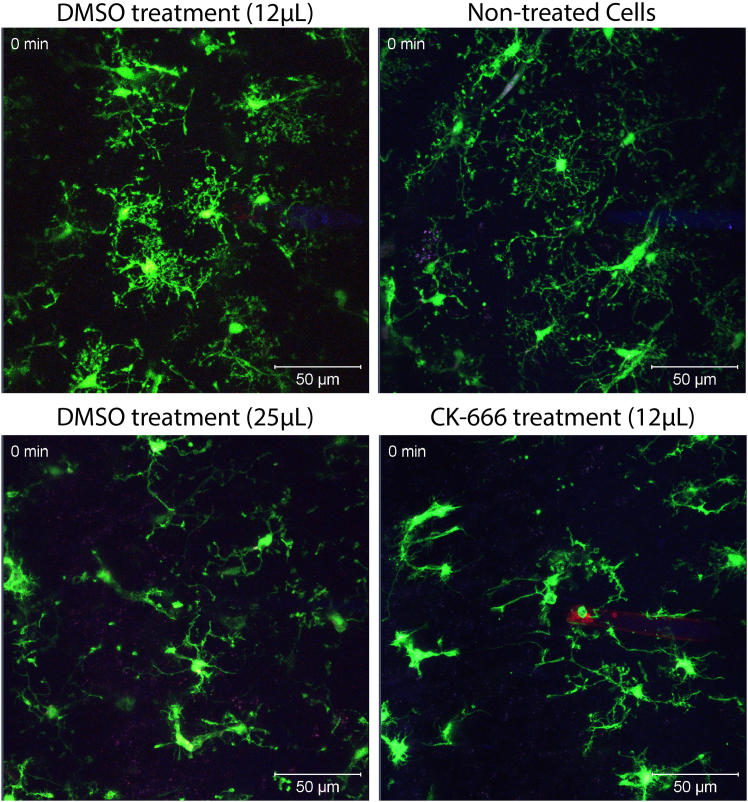
Troubleshooting – example of vehicle control impacting cell viability Pictures demonstrate our vehicle control, DMSO, at two different volumes (that matched the volume present in paired CK-666 experiments), as well as an example of non-treated cells. Images taken at the lower DMSO concentration match process arborization and cell morphology of non-treated cells. However, higher concentrations of DMSO result in cells looking more like the drug treated cells already at the beginning of acquisition. If, for a given mouse, cells in a non-treated tissue slice look normal but then do not in the vehicle control points towards the need to prepare a more concentrated drug to minimize vehicle volume. Scale bars represent 50 μm.

Cells in the vehicle control treatment injection seem to look unhealthy or appear dying, but the cells in the non-injection control and the drug injection seem just fine (after Step 27) (Figure 11).

### Potential solution

Vehicle controls can also be cytotoxic at high concentrations that may not be visible when the drug is present. For example, we found that 25 μL of DMSO per 10 mL of ACSF was cytotoxic to the cells. To lower the amount of vehicle control present, dissolve the drug in a higher stock concentration to ensure the least amount of vehicle is used to preserve cells. If possible, always do a non-treated injection (with neither vehicle nor drug component) to ensure the cells look good and to have a control for comparison, ensuring that vehicle is not causing any issues that would affect drug analysis. For example, we found that DMSO should be diluted at least 1:1000 to avoid cytotoxicity.

### Problem 14

Drug concentration does not appear strong enough to cause an effect or does not seem to last the entire length of the 2-hour video (after Step 27).

### Potential solution

*In vitro* cell culture can allow for establishing a baseline of what cells should look like with drug treatment (in terms of morphology and functional changes) but may misinform in terms of drug concentration. A pilot experiment with multiple runs at increasingly higher concentrations of drug may be helpful to verify desired effect.

We found that the injection pocket does not wash out (independent of bath flow rate), so dispersion of the injection volume amongst the cells is going to be local and not a time sensitive event. We also found that injecting the drug into the bath solution did not cause the cells to have any influence from the drugs present, and that washing over the cells with the drug did not equate to drug internalization.

For our experiments, in vitro cell culture pointed us towards a 125 μM CK-666 concentration, but we ended up needing to do a 200 μM concentration for our brain injections to see a similar response both morphologically and functionally in the *in situ* brain slice microglia.

### Problem 15

**Figure 12:**
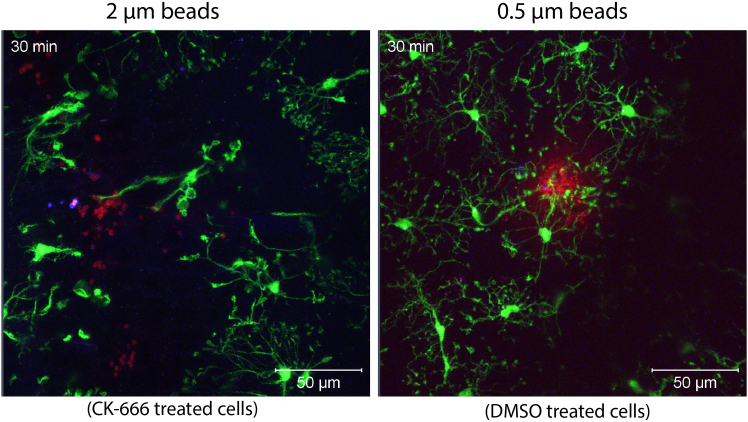
Troubleshooting – example of bead size differences and dispersion radius when injected into the tissue Scale bars represent 50 μm.

The cells don’t seem to be interacting with the beads (after Step 27) (Figure 12).

### Potential solution

Bead size could be an issue in signaling cells or alerting processes of the bound ligand cue. If cells do not seem stimulated to respond, a larger bead size may be necessary to ensure cell interaction. When comparing 0.5 μm iC3b-opsonized beads and 2 μm iC3b-opsonized beads, we found the cells only interacted with the 0.5 μm beads when they clustered large enough to measure roughly a 2 μm patch. We also found when injecting that the 2 μm beads dispersed further and allowed more cells to be measured interacting compared to the small pocket of beads that resulted from injecting 0.5 μm beads.

### Problem 16

**Figure 13:**
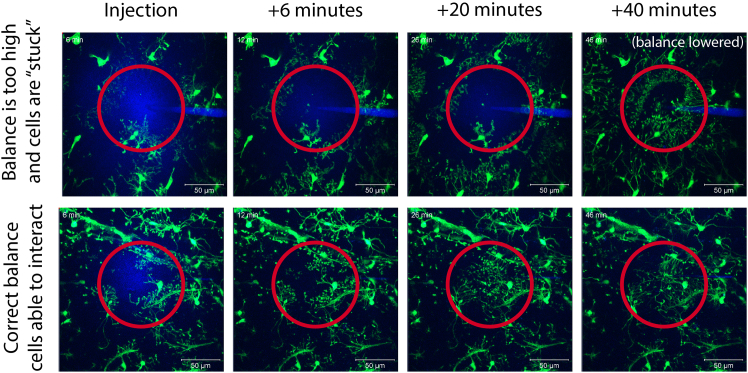
Troubleshooting – example of too much balance pressure on micropipette preventing cell interaction near micropipette tip Examples of a chemotaxis assay where ATP was injected alongside the blue Dextran, with cells that were imaged for an hour to watch cell processes extend to interact with the tip of the micropipette. As can be seen in the top line, too much balance pressure results in a steady deposition of dextran (the blue does not disappear), and processes do not close in on the tip of the micropipette as they do in the bottom row of images (amount of green fluorescence visible within the red circle surrounding the tip of the micropipette). Scale bars represent 50 μm.

After injection, blue Dextran persists at high intensity levels (does not wash out), and cells are unable to move closer to the injection spot, either to interact with the micropipette tip or beads present (after Step 27) (Figure 13).

### Potential solution

The balance pressure setting on the Pico spritzer is too high, and therefore the contents of the micropipette continue to be ejected into the tissue despite the actual timed injection having finished. Turn down or turn off the balance pressure of the micropipette and observe if the dextran intensity lowers. This can be done visually or via the region of interest intensity graph.

## Resource availability

### Lead contact

Requests for further information and resources should be directed to and will be fulfilled by the lead contact, Jeremy D. Rotty (jeremy.rotty@usuhs.edu).

### Technical contact

Technical questions on executing this protocol should be directed to and will be answered by the technical contact, Jeremy D. Rotty (jeremy.rotty@usuhs.edu).

### Materials availability

This study did not generate new unique reagents.

### Data and code availability

This study did not generate or analyze new datasets or code.

## Acknowledgments

We thank Dr. Zygmunt Galdzicki for the use of his lab’s vibratome and the Biomedical Instrumentation Center for use of the Zeiss LSM 7MP two-photon microscope. This work was supported by a 10.13039/100008544Cosmos Club Foundation award (to S.G.P.), a Uniformed Services University graduate student research award (to S.G.P.), and by the 10.13039/100000002National Institutes of Health (GM134104 to J.D.R.) and the 10.13039/100000005Department of Defense (HU00012320103 to J.D.R.), as well as startup funds from the 10.13039/100007188Uniformed Services University (to J.D.R.). This project is sponsored by the 10.13039/100007188Uniformed Services University of the Health Sciences (USU); however, the information or content and conclusions do not necessarily represent the official position or policy of, nor should any official endorsement be inferred on the part of USU, the Department of War, or the U.S. Government. The 10.13039/100007188Uniformed Services University of the Health Sciences (USU), 4301 Jones Bridge Rd., A1040C, Bethesda, MD 20814-4799, is the awarding and administering office.

## Author contributions

S.G.P.: experiments and planning and writing and editing. F.W.L.: experiments and planning and writing and editing. J.D.R.: project oversight, experiments and planning, writing, editing, and funding. All authors had the opportunity to review and comment on the manuscript prior to submission.

## Declaration of interests

The authors declare no competing interests.
